# Rho-Associated Protein Kinase Inhibitor Treatment Promotes Proliferation and Phagocytosis in Trabecular Meshwork Cells

**DOI:** 10.3389/fphar.2020.00302

**Published:** 2020-03-17

**Authors:** Wenshi Chen, Xuejiao Yang, Jingwang Fang, Yuqing Zhang, Wei Zhu, Xian Yang

**Affiliations:** ^1^Department of Ophthalmology, The Affiliated Hospital of Qingdao University, Qingdao, China; ^2^Department of Pharmacology, School of Pharmacy, Qingdao University, Qingdao, China; ^3^Department of Ophthalmology, Qingdao Central Hospital, Qingdao University, Qingdao, China

**Keywords:** Rho-associated protein kinase inhibitor, glaucoma, trabecular meshwork, proliferation, phagocytosis

## Abstract

**Purpose:**

Continuous reductions in trabecular meshwork (TM) cellularity inhibit aqueous humor (AH) outflow, which is the main cause of primary open-angle glaucoma. Rho-associated protein kinase inhibitor (ROCKi) targets the TM to reduce intraocular pressure (IOP) and increase AH outflow facility. However, the underlying mechanisms are not entirely clear. Here, we aimed to investigate the effect of a ROCKi (Y-27632) on TM cell proliferation and phagocytosis.

**Methods:**

Immortalized human TM (iHTM) cells, glaucomatous TM (GTM_3_) cells, and primary human TM (pTM) cells were cultured and identified. The effects of various concentrations of Y-27632 on F-actin cytoskeleton were assessed using immunofluorescence. Cell proliferation effects were evaluated using a cell counting kit-8 (CCK8), cell counting, and Ki67 immunostaining. Cell phagocytosis was evaluated using immunofluorescence and flow cytometry in immortalized TM cells. C57BL/6J and Tg-*MYOC*^Y437H^ mice were used to investigate the proliferative effects of Y-27632 on TM cells *in vivo*. The effect of Y-27632 on IOP was monitored for 2 weeks, and the outflow facility was detected 2 weeks after IOP measurement. TM cells in mice were counted using immunohistochemistry.

**Results:**

Y-27632 (100 μM) significantly promoted the proliferation of both immortal TM cells and pTM cells. In GTM_3_ cells, phagocytosis was significantly greater in the Y-27632 group than in the control group, nearly reaching the level of phagocytosis in iHTM, as determined using immunofluorescence and flow cytometry. In Tg-*MYOC*^Y437H^ mice, treatment with Y-27632 significantly decreased IOP and increased outflow facility, which greatly influenced the long-term IOP-lowering effect. The number of TM cells in Tg-*MYOC*^Y437H^ mice was significantly improved after Y-27632 administration.

**Conclusion:**

Y-27632 promoted cell proliferation and phagocytosis of TM cells, and its proliferative effect was demonstrated in a transgenic mouse model. These results revealed a new IOP-lowering mechanism of Y-27632 through effects on TM cells, suggesting the potential for a correlation between TM cellularity and long-term recovery of IOP.

## Introduction

Glaucoma is the leading cause of irreversible blindness worldwide, and pathological intraocular pressure (IOP) is the main underlying pathogenic factor. Normal IOP is regulated by a balance between the generation and drainage of aqueous humor (AH), and IOP is a function of aqueous humor formation (or flow) rate and resistance to drainage, as well as uveoscleral outflow rate and episcleral venous pressure (Millar et al., 2011; Jonas et al., 2017). Trabecular meshwork (TM) cells are the primary cells in the conventional outflow pathway of AH (Stamer and Clark, 2017). However, the cellular density of TM is reduced because of aging and glaucoma (Zhu et al., 2016). Accelerated loss of TM cells hinders the activity of the conventional outflow pathway, leading to increased IOP via a decreased outflow facility, which is the main risk factor for primary open-angle glaucoma (POAG) (Stamer et al., 2015).

Rho kinases (ROCKs) are serine/threonine kinases that are activated by interaction with Rho GTPases (Cherfils and Zeghouf, 2013). Although the functions of ROCKs are not entirely understood, the Rho/ROCK signaling pathway has been confirmed to participate in many processes, such as cell contraction, adhesion, migration and invasion, transformation, phagocytosis, and proliferation (Riento and Ridley, 2003). An important study demonstrated that the Rho/ROCK signaling pathway plays an essential role in TM function, AH outflow, and IOP (Rao et al., 2017).

Rho-associated protein kinase inhibitor (ROCKi) is a new class of IOP-reducing medicines that directly targets TM cells (Honjo and Tanihara, 2018). Two types of ROCKi are approved for clinical use for treating glaucoma, including ripasudil (K-115) in Japan and netarsudil in the United States (Garnock-Jones, 2014; Sturdivant et al., 2016, Tanna and Johnson, 2018). ROCKi has been shown to increase TM cell relaxation (Gong and Yang, 2014; Li et al., 2016); reduce actin crosslinking (Honjo et al., 2001; Inoue and Tanihara, 2013), cell adhesive interactions, and cell tension (Shen et al., 2008; Pattabiraman et al., 2014); and decrease transdifferentiation and fibrogenic rigidity (Pattabiraman et al., 2014). Notably, these functional changes increase AH outflow and decrease IOP (Rao et al., 2017). There are several additional effects of ROCKi that aid in glaucoma treatment, including increased retinal blood flow, extended axonal length (Sugiyama et al., 2011), improved retinal ganglion cell (RGC) survival (Yamamoto et al., 2014), and wound healing regulation (Honjo et al., 2007).

Recently, ROCKi was shown to promote the proliferation of corneal endothelial cells (CECs) (Alvarado et al., 1981). In the development of human eyes, TM cells and CECs both originate from neural crest cells (Tian et al., 2012). Therefore, we hypothesized that ROCKi also promote the proliferation of TM cells. A recent study found that RKI-1447, a ROCKi, significantly reduces IOP by increasing TM phagocytosis, which is an essential function of TM cells (Dang et al., 2019). Y-27632 is the first ROCKi drug reported to reduce IOP (Honjo et al., 2001), and its IOP-lowering effect was verified in multiple species (Gong and Yang, 2014). However, to date, no study has elucidated on the role of Y-27632 in phagocytosis.

The present study was designed to determine whether Y-27632, a ROCKi, can improve TM cell proliferation both *in vitro* and *in vivo*, as well as phagocytosis *in vitro*; it was also designed to determine how Y-27632 affects the IOP and AH outflow facility. Our results suggest that the ROCKi Y-27632 promotes TM cell proliferation and phagocytosis and significantly reduces long-term IOP and increases AH outflow. These results provide insights into the mechanism of IOP reduction by ROCKi.

## Materials and Methods

### Materials

Dulbecco’s modified Eagle’s medium (DMEM)/F-12 medium and Minimum Essential Medium (MEM)-alpha medium were purchased from Gibco (Grand Island, NY, United States). Cell counting kit-8 (CCK8) for cell proliferation was purchased from Dojindo (Kumamoto, Japan). Y-27632 was purchased from EMD Millipore (Darmstadt, Germany). Polyclonal anti-Ki67 antibody, Alexa Fluor^TM^ 568 goat anti-rabbit IgG and Alexa Fluor^TM^ 488 goat anti-mouse IgG, phalloidin, and 4′,6-diamidino-2-phenylindole (DAPI) were purchased from Novusbio (Littleton, CO, United States), Invitrogen (Carlsbad, CA, United States), Abcam (Cambridge, MA, United States), and Santa Cruz (Dallas, TX, United States), respectively.

### Cell Culture

Primary human TM (pTM) cells were obtained from the Lion Eye Bank (Iowa, IA, United States). The isolated pTM cells were cultured in MEM-alpha medium with 10% fetal bovine serum (FBS; Hyclone, Logan, UT) and 0.2% primocin (InvivoGen; San Diego, CA, United States). Immortalized human TM (iHTM) cells were kindly provided by Dr. Vincent Raymond (Laboratory of Ocular Genetics and Genomics; Quebec City, Canada), and glaucomatous human TM (GTM_3_) cells were gifted by Prof. Yuhao Peng (Glaucoma Research, Alcon Laboratory; Fort Worth, TX, United States). All cell lines were cultured in DMEM/F-12 medium containing 15% FBS (Every green; Zhejiang, China) and 1% penicillin–streptomycin (Gibco) and were maintained at 37°C with 5% CO_2_ in a humidified tissue culture incubator.

### Immunofluorescence

Cells were cultured on poly-D-lysine–coated coverslips (Solarbio; Beijing, China) and fixed using 4% paraformaldehyde (Thermo; Waltham, MA, United States) at room temperature (RT) for 15 min. The cells were then rinsed in Dulbecco’s phosphate-buffered saline (DPBS) for 5 min. The coverslips were incubated in a blocking solution (1% bovine serum albumin with 0.3% Triton X-100) (Sigma; St. Louis, MO, United States) for 1 h and then incubated overnight at 4°C with rabbit polyclonal anti-matrix metallopeptidase 3 (MMP3) antibody (1:100; Abcam), rabbit polyclonal anti-tissue inhibitor of metalloproteinase 3 (TIMP3) antibody (1:100; Abcam) and mouse monoclonal anti-collagen IV (COL IV) antibody (1:100; Abcam). Subsequently, coverslips were incubated with Alexa Fluor^TM^ 568 goat anti-rabbit IgG and Alexa Fluor^TM^ 488 goat anti-mouse IgG secondary antibodies for 1 h at RT.

For cytoskeletal staining, various concentrations of Y-27632 were added to cultured iHTM and GTM_3_ for 4 h on coverslips and fixed with 4% paraformaldehyde. After washing with DPBS three times, 0.3% Triton X-100 was added to increase permeability. The coverslips were then incubated with phalloidin (1:1000; in blocking solution) for 1 h at RT. Coverslips were washed three times in DPBS, and the nuclei of TM cells were stained with DAPI at RT for 15 min.

### Western Immunoblotting

TM cells cultured in a 6-well plate were treated with 0.1% ethanol (ETH) or 100 nM dexamethasone (DEX; Sigma) for 5 days. After washing with DPBS, TM cells were lysed RIPA (Thermo). Protein concentration was measured using the BCA Protein Assay Reagent Kit (Thermo). Total protein (50 μg) was mixed with 4x LDS sample buffer (Invitrogen) and boiled for 5 min before loading. Proteins were separated using 8% sodium dodecyl sulfate–polyacrylamide gel electrophoresis and then transferred to polyvinylidene fluoride membrane using Mini *Trans*-Blot Cell (Bio-rad; Hercules, CA, United States) under 150 mA for 1.5 h. The membrane was blocked with 5% dry milk and probed with rabbit anti-myocilin antibody (1:500; Abcam) and rabbit GAPDH antibody (1:10000; Abcam) overnight at 4°C. Next, the membrane was incubated with goat anti-rabbit secondary antibody conjugated with HRP (1:10000; Abcam). Singles were visualized under chemiluminescent detection with Super Signal^TM^ West Pico PLUS Chemiluminescent Substrate (Thermo) in a chemidoc XRS^plus^ imaging system (Bio-Rad). Band intensity was analyzed using Image Lab software (Bio-Rad).

### Cell Proliferation Assay

To investigate the effects of Y-27632 on the proliferation of iHTM and GTM_3_ cells, various concentrations of Y-27632 were added to cultured iHTM and GTM_3_ cells for 24 h; the cells were then assayed using the CCK-8 kit in accordance with the manufacturer’s instructions. Briefly, cultured TM cells were suspended in the culture medium and seeded in a 96-well plate (5 × 10^3^ cells/well) along with 0, 10, 30, 100, or 200 μM of Y-27632. After 24 h of incubation, 10 μL of CCK-8 was added to each well of the plate. The plate was incubated for an additional 2 h, and the absorbance at 450 nm was measured using a microplate reader (Tecan; Switzerland).

### Cell Counting

PTM cells were cultured with 100 μM of Y-27632 in a 6-well plate (1 × 10^5^ cells/well). After 24 or 48 h of incubation, cells were washed twice in DPBS and briefly treated with 0.5 mL of TrypLE^TM^ Express (Gibco) for 3 min; 1 mL of medium was added to each well to stop digestion, and each cell suspension was transferred to a 1.5 mL tube. The tubes were vortexed, and 15 μL of each cell suspension was combined with 15 μL of trypan blue (Solarbio). Subsequently, 20 μL of this mixture was added to the cell counting plate, and each well was consecutively counted three times using a cell counter (Counter star; Shanghai, China).

### Ki67 Immunostaining

The Ki67 marker was used to evaluate the proliferation of pTM cells exposed to Y-27632. PTM cells were seeded on coverslips in 24-well plates (1 × 10^3^ cells/well) and incubated with or without 100 μM Y-27632 for 24 or 48 h. Cells were fixed in 4% formaldehyde for 15 min at RT, washed with DPBS, and permeabilized with 0.3% Triton X-100 in DPBS. Cells were then incubated in the blocking solution for 1 h. Samples were stained with rabbit polyclonal anti-Ki-67 antibody (1:200; Novusbio) at 4°C overnight, washed with DPBS, and incubated with Alexa Fluor^TM^ 568 goat anti-rabbit IgG (1:200) for 1 h at RT. Cells were counterstained with DAPI to stain the nuclei. The stained cells were then photographed using a confocal microscope (Nikon; Tokyo, Japan).

### Measurement of Phagocytosis

To assess phagocytosis, iHTM and GTM_3_ were seeded on 35 mm cell culture dishes for immunofluorescence or in 6-well plates for flow cytometry analysis (FACS). They were incubated with 100 μM of Y-27632 and normal medium for vehicle control groups. After 24 h of incubation, fluorescent beads (Invitrogen) 1 μm in diameter were added and cells were incubated for an additional 2 h. Cells were then washed three times in DPBS and photographed using a confocal microscope or resuspended in 1ml of DPBS and detected with a FACSCalibur flow cytometer (BD; San Jose, CA, United States) using CellQuest software in FITC channel. The fluorescence intensity was analyzed using NIS-eliments (Nikon; Tokyo, Japan) or FlowJO-V5 (San Francisco, CA, United States).

### Mice

Six-month-old male C57BL/6J mice and 8–10 months male myocilin transgenic mice with Y437H mutation (Tg-*MYOC*^Y437H^ on a C57BL/6J background) were used to measure IOP and outflow facility, as well as for immunohistochemical analysis. The mice were maintained in standard housing at 22°C with a 12 h light/12 h dark cycle (lighting from 6:00 am to 6:00 pm) and controlled humidity. All the animal experimental procedures were approved by the Qingdao University Medical Center and followed institutional guidelines for laboratory animal care and use.

### Subconjunctival Injection

Mice were sedated with 2.5% isoflurane before surgery. The assistant held the eyelids apart, and the operator lifted the bulbar conjunctiva with fine forceps, 100 μM of Y-27632 was delivered into the bulbar subconjunctival space using a 33-gauge needle (Becton Dickenson) under an operating microscope. Formation of a subconjunctival bleb ensured successful injection. The vehicle control group comprised mice that received an equal amount of 0.9% saline. To explore the effects of Y-27632 on IOP, mice received daily subconjunctival injections for 4 days in different quadrants.

### IOP Measurements

Mice were anesthetized by gas anesthesia with 2.5% isoflurane plus 80% (vol/vol) oxygen. All IOPs were measured using a rebound tonometer (TonoLab; Helsinki, Finland) between 11 PM and 12 AM with minimum red-light illumination. All measurements were recorded 5 min after induction of anesthesia. The IOP was measured three times per eye, and the average value was calculated for each eye; the IOP was tracked for 2 weeks at the same time every day.

### Outflow Facility Measurement

**FIGURE 1:**
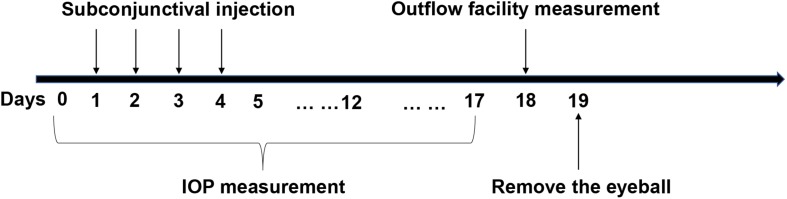
The specific operational procedures of the animal experiments. Y-27632 was delivered via subconjunctival injection for four consecutive days. Intraocular pressure (IOP) was detected from the time before administration to 2 weeks after injection. Outflow facility was measured in 2 weeks subsequently and then the eyeballs were removed for the follow up experiments.

As previously described (Zhu et al., 2016), mice were anesthetized via an intraperitoneal injection of 8% chloral hydrate followed by cannulation of the anterior chamber with a 33-gauge needle. A 150 μL Hamilton syringe (Hamilton, United States) was mounted on a computer-controlled syringe pump (World Precision Instruments; FL, United States), and 0.9% saline was pumped into the eye. The pressure in the system was maintained by a flow-through pressure transducer (IcuMedical; San Clemente, CA, United States). Outflow facility (μL/min/mmHg) was calculated using the HemoLab software (Stauss Scientific; Iowa City, IO, United States) to determine flow rates (μL/min) for sustaining pressures of 15, 25, and 35 mmHg. Figure 1 is a detailed schematic of the animal experiments.

### Immunohistochemistry

To investigate the proliferation of TM cells in ROCKi-treated tissue, we assessed the effect of Y-27632 on Tg-*MYOC*^Y437H^ mice. Murine eyes were embedded in Optimal Cutting Temperature Compound (Sakura; Tokyo, Japan), frozen, and sectioned to 8-μm thickness on a cryostat equipped with a tape transfer system (CryoJane, Leica). Samples were fixed in 95% ETH for 5 min and rinsed in DPBS for 5 min. Triton X-100 (0.3%) in DPBS was added to increase the permeability for 5 min; samples were then washed twice with DPBS. Samples were incubated with phalloidin (1:1000) in the blocking solution for 1 h at RT. Samples were then washed three times and incubated with DAPI for 20 min at RT. After an additional wash with DPBS, coverslips were mounted on the samples by using Neutral Balsam (Solarbio), and images were obtained using confocal microscopy.

### Statistical Analysis

Statistical analyses were performed using GraphPad Prism (GraphPad Inc; San Diego, CA, United States). Data were expressed as the mean ± standard error of the mean and compared using unpaired *t*-tests, one-way ANOVA with Dunnett’s test and two-way ANOVA with Bonferroni post-tests. *P* values less than 0.05 were considered significant. The investigators who counted the number of cells were blinded to which group the sample belonged to.

## Results

### Characterization of Human TM Cells

**FIGURE 2:**
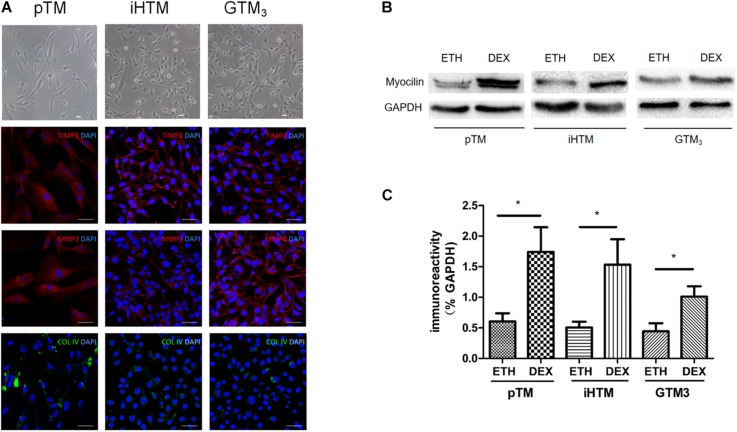
Characterization of primary human trabecular meshwork (pTM) cells and immortal trabecular meshwork (TM) cells. **(A)** The morphology of pTM, immortal human trabecular meshwork cells (iHTM) and glaucomatous human trabecular meshwork cells (GTM_3_) in observed by phase contrast microscope. Positive staining of biomarkers, including TIMP3 (red), MMP3 (red) and COL IV (green) for TM cells. Cell nuclei were stained with DAPI (blue). Bar = 50 μm. **(B)** Effect of dexamethasone (DEX) for 5 days on induced the expression of myocilin in pTM, iHTM and GTM_3_ cells. **(C)** Intensity of visualized bands of myocilin proteins in control and DEX-treated cells from pTM and immortal TM cells. The results were quantified from three independent experiments (*n* = 3) by Image Lab software, and the expression of myocilin proteins was significantly higher in these TM cells after DEX treatment by unpaired *t*-tests. *p < 0.05.

Primary and immortal TM cells in medium were photographed using microscopy. Immunofluorescence staining revealed that both primary and immortal TM cells expressed TM biomarkers, including MMP3, TIMP3 and COL IV proteins (Figure 2A). The staining of negative control group can be seen in Supplementary Material. We also compared the expression of myocilin, a glucocorticoid-inducible gene in the TM cells. Western blot showed the expressions of myocilin in primary and immortal TM cells were increased after DEX treatment (Figure 2B) and the intensity of the visualized bands illustrated that DEX induced the expression of myocilin (^∗^*p* < 0.05, Figure 2C). Cell morphology, immunofluorescence analysis, and western blot confirmed that these cell lines and isolated cells from human TM tissue had characteristics of TM cells.

### Y-27632 Modulated Cytoskeleton Characteristics and Promoted the Proliferation of iHTM Cells and GTM_3_ Cells *in vitro*

**FIGURE 3:**
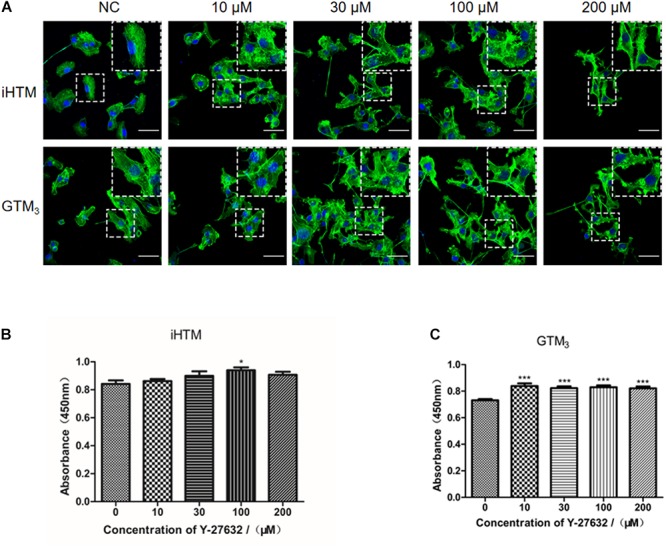
Effect of different concentrations of Y-27632 on cytoskeleton (F-actin) and cellularity in iHTM cells and GTM_3_ cells. **(A)** Immunofluorescence staining of F-actin (green). The nuclei were counterstained with DAPI (blue). The amplified part of the figures was in the upper right corner. Bar = 50 μm. **(B,C)** Cell proliferation was analyzed using CCK-8 assay (*n* = 6 independent replicate experiments). Statistical analyses were performed using one-way ANOVA with Dunnett’s test. *p < 0.05 and ***p < 0.001.

Treatment with Y-27632 (10, 30, 100, and 200 μM) resulted in dose-dependent changes in the levels of decreased actin stress fibers, as shown by relative staining of F-actin (phalloidin) in iHTM and GTM_3_ cells (Figure 3A). CCK8 analysis of iHTM cells revealed that treatment with 100 μM of Y-27632 caused a significant increase in cell number (^∗^*p* < 0.05, Figure 3B), whereas the CCK8 analysis of GTM_3_ cells revealed that treatment with all tested concentrations of Y-27632 caused significant increases in cell number compared with the control condition (^∗∗∗^*p* < 0.001, Figure 3C).

### Y-27632 Promoted the Proliferation of pTM Cells *in vitro*

**FIGURE 4:**
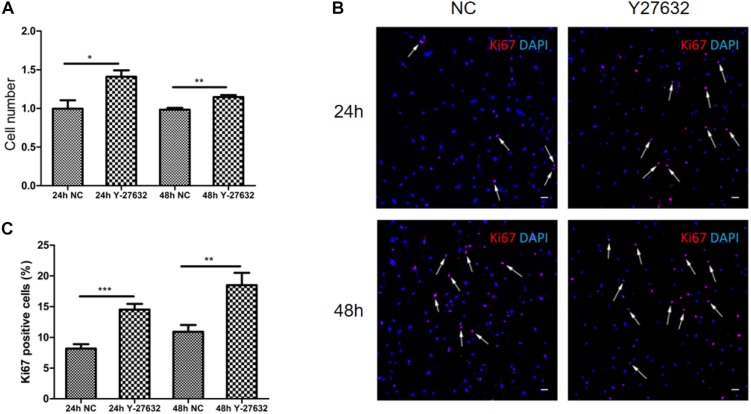
Y-27632 promoted the proliferation of pTM cells. **(A)** The cell numbers of pTM cells incubated with 100 μM of Y-27632 for 24 and 48 h. Three independent experiments were carried out (*n* = 3). Bar = 50 μm. **(B)** Immunofluorescent staining was performed with anti-Ki67 antibody (red). The nuclei were stained with DAPI (blue). **(C)** The percentage of Ki67-positive pTM cells (pink fluorescence in nuclei, arrows highlight several, but not all). Three random fields in each three coverslips were counted. Data were analyzed by unpaired -*t* test. *p < 0.05, **p < 0.01, and ***p < 0.001.

Treatment with Y-27632 significantly increased the number of pTM cells (^∗^*p* < 0.05 and ^∗∗^*p* < 0.01, Figure 4A). The effect of Y-27632 was more obvious after 48 h than after 24 h. As shown in Figure 4B, positive Ki67 staining was present in a subset of pTM cells (pink), and the percentage of these positive cells was associated with the duration of treatment with Y-27632. The number of pTM cells that were positive for Ki67 was significantly greater than that in the control condition (^∗∗^*p* < 0.01 and ^∗∗∗^*p* < 0.001, Figure 4C).

### Y-27632 Promoted the Phagocytosis of GTM_3_ Cells

**FIGURE 5:**
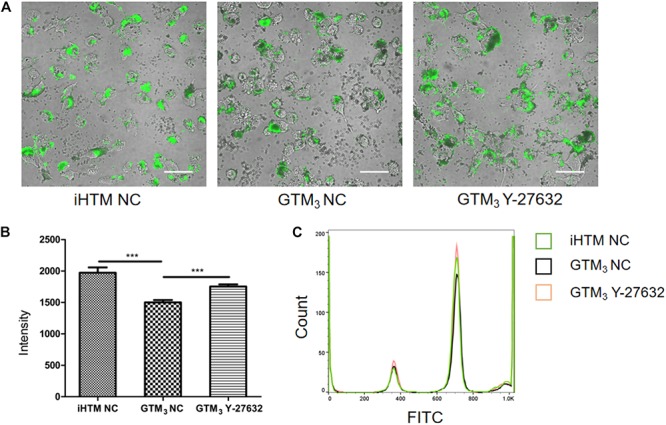
Y-27632 promoted phagocytosis of GTM_3_ cells. **(A)** Photograph of TM cells 2 h after exposure to fluorescent beads. Phagocytosed particles fluoresced green. Bar = 50 μm. **(B)** Phagocytosis was measured as fluorescence intensity per section by NIS-eliments and intensity was analyzed by one-way ANOVA with Dunnett’s test, **p < 0.01 and ***p < 0.001. **(C)** Histogram shows the relative expression level of FITC intensity in phagocytes of iHTM control group (green), GTM_3_ control group (black), and GTM_3_ Y-27632 group (orange).

As shown in Figure 5A, fluorescent beads (green) were present in the cytoplasm, and the fluorescence intensity significantly differed between Y-27632-treated and control GTM_3_ cells (^∗∗^*p* < 0.01 and ^∗∗∗^*p* < 0.001, Figure 5B); the control iHTM cells also showed higher fluorescence than the Y-27632-treated GTM_3_ cells. FACS analyses confirmed that the intensity of the Y-27632 group was higher than that of the control GTM_3_ group (Figure 5C). These results indicate that Y-27632 promotes the phagocytosis of GTM_3_ TM cells.

### Y-27632 Reduced IOP and Increased AH Outflow *in vivo*

**FIGURE 6:**
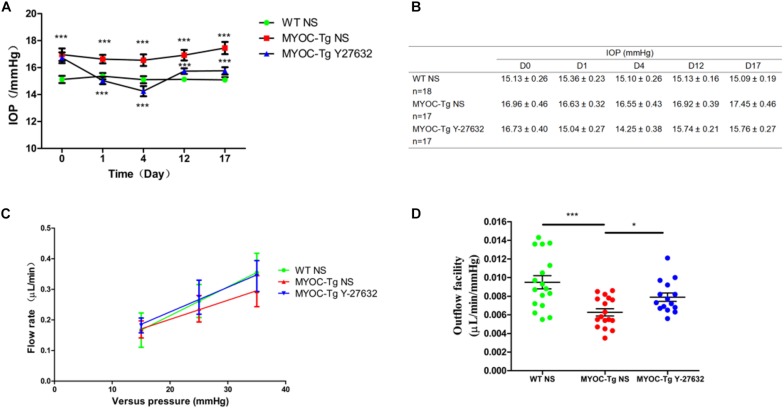
Y-27632 decreased the IOP and increased the aqueous humor (AH) outflow facility in mice. **(A)** The IOPs that were tracked daily after subconjunctival injection with Y-27632 or 0.9% saline. IOP in the wild-type (WT) control (*n* = 18) was significantly lower than in the MYOC-Tg control (*n* = 17). Compared with the IOP in MTOC-Tg control, the IOP was significantly decreased after treatments with Y-27632 (*n* = 17). IOPs were analyzed by two-way ANOVA with Bonferroni post-tests and ***p < 0.001. **(B)** The detailed IOP values of three groups before, during and after injection. **(C)** Linear relationship of versus pressures and flow rate in the three groups. **(D)** Outflow facilities that were measured at 2 weeks after injection. The outflow facility of the WT control (*n* = 17) is significantly higher than that in the MYOC-Tg control (*n* = 17). Compared with the average value of outflow facility in the MYOC-Tg control, an increased outflow facility was observed after Y-27632 treatment (*n* = 16). Data were analyzed using one-way ANOVA with Dunnett’s test, *p < 0.05.

The IOP did not significantly differ between Y-27632–treated and control Tg-*MYOC*^Y437H^ mice before subconjunctival injection, however, the IOP was higher in both groups of transgenic mice than in wild-type mice (^∗∗∗^*p* < 0.001, Figure 6A). Transgenic mice that received subconjunctival injection of 100 μM of Y-27632 exhibited significantly lower IOPs than the control transgenic mice (^∗∗∗^*p* < 0.001). Lower IOP was sustained with daily subconjunctival injection of Y-27632. After injection, the IOP slightly increased but remained lower than that in control transgenic mice (^∗∗∗^*p* < 0.001). Specific IOP values are detailed in Figure 6B.

Figure 6C showed the relationship between pressures (15, 25 and 35 mmHg) and flow rate in wild-type mice and transgenic mice. The slope represents outflow facility, and the results are shown in Figure 6D. There was a significant difference in outflow facility between wild-type and Tg-*MYOC*^Y437H^ mice (^∗∗∗^*p* < 0.001), indicating the successful establishment of the glaucomatous animal model. The AH outflow facility after Y-27632 treatment (0.0079 μL/min/mmHg) was significantly increased compared with that observed in vehicle control-injected transgenic mice (0.0063 μL/min/mmHg, ^∗^*p* < 0.05).

### Y-27632 Promoted the Proliferation of TM Cells *in vivo*

**FIGURE 7:**
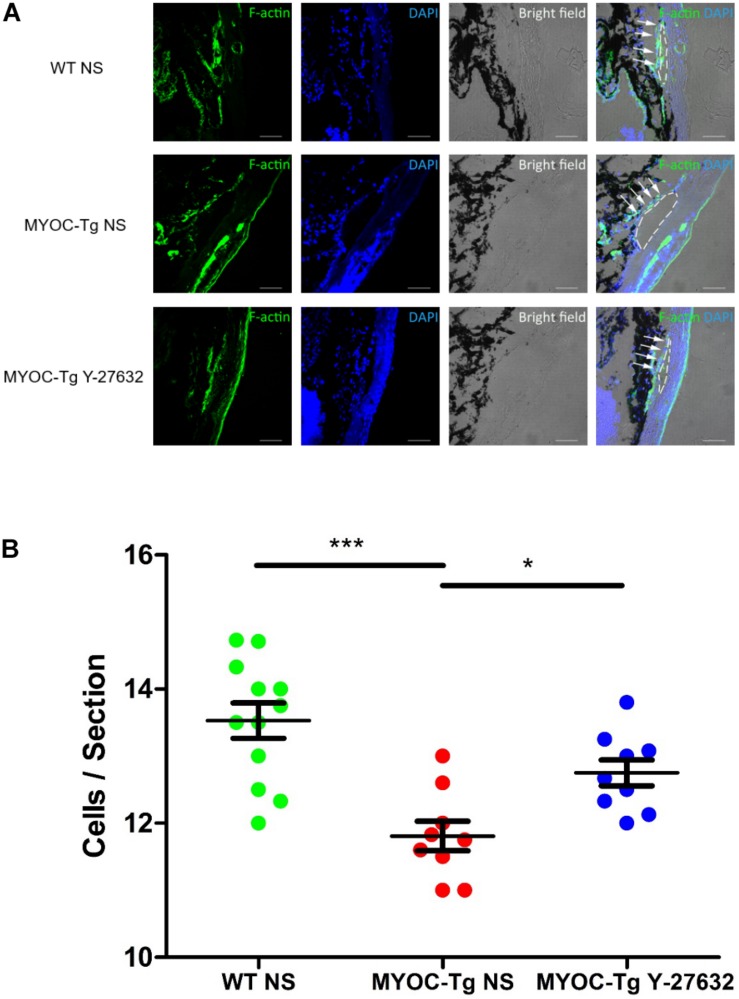
Y-27632 promoted the proliferation of TM cells in mice model. **(A)** F-actin (green) was localized in TM, as shown by immunofluorescence staining. Cell nuclei were stained with DAPI (blue). The TM cells (not all) were labeled by white arrows and Schlemm’s canals were marked by white dotted circles. Bar = 50 μm. **(B)** The number of TM cells in the MYOC-Tg control (*n* = 9) significantly decreased compared with that in the WT control (*n* = 12). The number of TM cells in the MYOC-Tg Y-27632 (*n* = 9) was significantly increased compared with those in the MYOC-Tg control. TM cells from six sections were counted and the numbers were averaged. Data were analyzed by one-way ANOVA with Dunnett’s test, *p < 0.05 and ***p < 0.001.

Phalloidin labeling of F-actin (green) in TM tissue was strongly positive in C57BL/6 mice compared with transgenic mice; treatment with Y-27632 improved the intensity of phalloidin labeling in transgenic mice (Figure 7A). Quantitative comparison showed a significant difference in the number of TM cells between transgenic and wild-type mice (^∗∗∗^*p* < 0.001, Figure 7B). The increased number of cells in the Y-27632-treated mice confirmed that Y-27632 treatment promoted the proliferation of TM cells.

## Discussion

The only proven effective method for the treatment of POAG is the reduction of IOP. If IOP is decreased by 20–40%, the average rate of progressive visual field loss is reduced by half (Quigley, 2011; Jonas et al., 2017). Topical monotherapy remains the first choice of treatment, and prostaglandin analogs are the first-line medicines in POAG (Dikopf et al., 2017; Jonas et al., 2017). The new IOP-lowering ROCKi (K-115) exhibit effects more potent than those of 0.005% latanoprost in monkeys, although only a few countries permit the use of ROCKi drugs in clinical practice (Dikopf et al., 2017). In this study, we found that treatment with the ROCKi (Y-27632) promoted proliferation and phagocytosis of TM cells. In addition, Y-27632 showed long-term effects of lowering IOP and promoting outflow facility, which suggests that the mechanism includes changes in the TM cell cytoskeleton, as well as increased TM cell proliferation and phagocytosis.

Y-27632 was previously reported to induce morphologic changes and reorganize the cytoskeleton in pTM cells, which increased AH outflow facility (Rao et al., 2001). In our study, we found that Y-27632 could also reorganize the cytoskeleton of immortal TM cell lines, as evidenced by changes in immunofluorescence. CCK8 analysis showed that various concentrations of Y-27632 promoted the proliferation of GTM_3_ cells, but only 100 μM of Y-27632 promoted the proliferation of iHTM cells; thus, ROCKi may be more effective in pathological glaucomatous cells, and the specific mechanism deserves further investigation.

In eye development, TM cells and CECs are derived predominantly from the neural crest (Tian et al., 2012); both cell types also have some attributes of endothelial lining cells, which may be related to structural integrity and outflow pathway fluency. TM cellularity decreases with age; CEC cellularity exhibits a similar progression (Alvarado et al., 1981). A previous study found that ROCKi promotes CEC proliferation, and the investigators suggested that ROCKi is negatively involved in the proliferation of CECs via phosphoinositide 3-kinase signaling (Okumura et al., 2014). In our study, we found that ROCKi also has a proliferative effect on TM cells.

We found that the effect of Y-27632 on the proliferation of pTM cells increased with exposure time, as determined by cell counting and staining for the proliferation marker Ki67. Thus, we presume that the overall effects of Y-27632 increase with time. MYOC is the most commonly mutated gene in patients with POAG, and the pathogenic mechanism observed in adult Tg-*MYOC*^Y437H^ mice is similar to that observed in POAG; thus, these transgenic mice have been used as a model of POAG (Zode et al., 2011). In addition, we verified the ability of Y-27632 to promote TM proliferation in Tg-*MYOC*^Y437H^ mice. We found that the proliferative effect of ROCKi is associated with the long-term reduction of IOP and promotion of AH outflow. A recent clinical study assessed the 1-year follow-up outcomes of 0.4% ripasudil and revealed an IOP-lowering effect in both monotherapy and additive therapy (Tanihara et al., 2016). The methods of ROCKi delivery may affect the site and mechanism of action. Repeated topical applications can achieve delivery of ROCKi drugs to both anterior and posterior segments, however, intravitreal, subconjunctival, and suprachoroidal injection methods may be more effective for delivery to posterior segment components, such as RGCs and optic nerve axons (Isobe et al., 2016; Pitha et al., 2018). Thus, we used subconjunctival injection to target TM cells, RGCs, and optic nerve axons. Our study was initially focused on the use of short-term subconjunctival administration, but we found that an ocular hypotensive effect could be maintained for an extended period after discontinuation of treatment. This indicated that ROCKi could support the regeneration of abnormal GTM_3_ cells and enable the recovery of cell function, which should be confirmed in future studies. There is a positive relationship between elevated IOP and TM loss (Alvarado et al., 1984); we presume that treatment with the ROCKi lowered IOP through short-term changes to TM cell morphology, relaxation and adhesion. In addition, the recovery of normal IOP prevents further TM cell loss, whereas ROCKi treatment promotes cell proliferation; therefore, IOP is maintained at a normal level for an extended period.

TM cells have various functions, including synthesis and degradation of collagen, proteoglycans, and other extracellular matrix components, as well as phagocytosis of extracellular debris (Buller et al., 1990). When these functions are not performed sufficiently to maintain normal conditions, the essential role of phagocytosis in keeping TM channels free of obstructive debris is disrupted (Gagen et al., 2013). A previous study found that phagocytosis was disrupted in pathological TM cells, but that one type of ROCKi (RKI-1447) could recover TM phagocytosis to a near reference range (Dang et al., 2019). The results of immunofluorescence and flow cytometry analyses in our study indicate that Y-27632 has a similar effect in promoting phagocytosis by GTM_3_ cells, such that it nearly recovered to the level observed in normal TM cells. This further illustrated the restorative function of ROCKi treatment on TM cells and showed that the IOP-reducing effect was also associated with TM phagocytosis.

In summary, the promotion of TM cell proliferation and phagocytosis by ROCKi treatment may be an underlying mechanism for long-term IOP reduction and AH outflow increase. A thorough investigation of the mechanisms by which proliferation and phagocytosis are promoted will improve the understanding of the therapeutic mechanism of ROCKi drugs. If ROCKi drugs are confirmed to promote proliferation and phagocytosis in the human body, they may have clear therapeutic effects on the main sites of glaucoma pathogenesis, which will broaden their application in the field of glaucoma and potentially change first-line drug choices for glaucoma treatment.

## Data Availability Statement

All datasets generated for this study are included in the article/Supplementary Material.

## Ethics Statement

The animal study was reviewed and approved by the Qingdao University Medical Center.

## Author Contributions

WC, XuY, JF, and YZ conducted experiments resulting in the data. WC, XuY, WZ, and XiY analyzed and interpreted the data. WZ and XiY conceived and designed the research and made critical revision of the manuscript for important intellectual content. XuY, WZ, and XiY obtained the funding and supervised research personnel.

## Conflict of Interest

The authors declare that the research was conducted in the absence of any commercial or financial relationships that could be construed as a potential conflict of interest.
