# Supplementary material for: Rho-Associated Protein Kinase Inhibitor Treatment Promotes Proliferation and Phagocytosis in Trabecular Meshwork Cells
Source: Front Pharmacol. 2020 Mar 17;11:302. doi: 10.3389/fphar.2020.00302 (PMC7090161; doi:10.3389/fphar.2020.00302)
Supplement: Supplementary file 1 [file Data_Sheet_1.pdf]

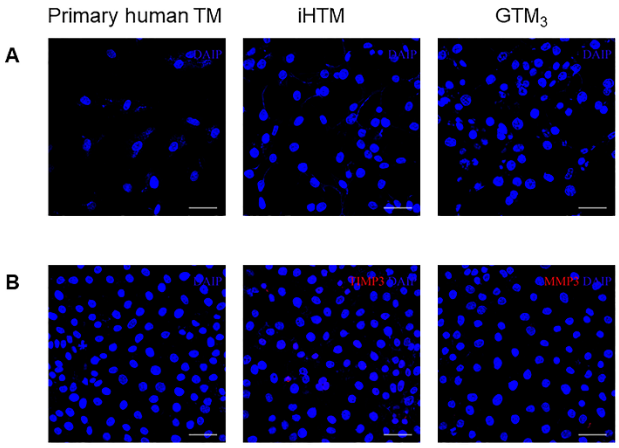

Figure S1. The control group Immunofluorescence staining of TM cell and TM protein in human corneal epithelial cells (HCEC)

A: The control group without primary antibodies. B: There are no expressions of TM biomarkers: MMP3 and TIMP3 in HCEC. Cell nuclei were stained with DAPI (blue). Bar=50 µm.
